# Relationship between Physical Activity, Oxidative Stress, and Total Plasma Antioxidant Capacity in Spanish Children from the GENOBOX Study

**DOI:** 10.3390/antiox10020320

**Published:** 2021-02-20

**Authors:** Francisco Jesús Llorente-Cantarero, Francisco Javier Aguilar-Gómez, Rosaura Leis, Gloria Bueno, Azahara I. Rupérez, Augusto Anguita-Ruiz, Rocío Vázquez-Cobela, María Dolores Mesa, Luis A. Moreno, Ángel Gil, Concepción María Aguilera, Mercedes Gil-Campos

**Affiliations:** 1 Department of Specific Didactics, Faculty of Education, Maimónides Institute of Biomedicine Research of Córdoba (IMIBIC), University of Córdoba, 14071 Córdoba, Spain; llorentefj@yahoo.es; 2 CIBEROBN, (Physiopathology of Obesity and Nutrition) Institute of Health Carlos III (ISCIII), 28029 Madrid, Spain; mariarosaura.leis@usc.es (R.L.); gbuenoloz@yahoo.es (G.B.); augustoanguita@ugr.es (A.A.-R.); cobela.rocio@gmail.com (R.V.-C.); lmoreno@unizar.es (L.A.M.); agil@ugr.es (Á.G.); mercedes_gil_campos@yahoo.es (M.G.-C.); 3 Metabolism and Investigation Unit, Reina Sofía University Hospital, Maimónides Institute of Biomedicine Research of Córdoba (IMIBIC), University of Córdoba, 14004 Córdoba, Spain; fjaviagui1992@gmail.com; 4 Unit of Investigation in Nutrition, Growth and Human Development of Galicia, Pediatric Department, University of Santiago de Compostela, Clinic University Hospital of Santiago, Instituto de Investigación Sanitaria de Santiago (IDIS), 15706 Santiago de Compostela, Spain; 5 Pediatric Endocrinology Unit, Clinic University Hospital Lozano Blesa, Facultad de Medicina, Universidad de Zaragoza, 50009 Zaragoza, Spain; 6 GENUD Research Group, Instituto Agroalimentario de Aragón (IA2), Instituto de Investigación Sanitaria (IIS) Aragón, University of Zaragoza, 50009 Zaragoza, Spain; airuperez@unizar.es; 7 Department of Biochemistry and Molecular Biology II, Institute of Nutrition and Food Technology “José Mataix”, Center of Biomedical Research, University of Granada, Armilla, 18016 Granada, Spain; mdmesa@ugr.es; 8 Instituto de Investigación Biosanitaria ibs Granada, 18014 Granada, Spain

**Keywords:** physical activity, accelerometry, oxidative stress, plasma total antioxidant capacity, 8-hydroxy-2′-deoxyguanosine, isoprostane F2α

## Abstract

The World Health Organization has recommended performing at least 60 min a day of moderate-to-vigorous physical activity (MVPA) and reducing sedentarism in children and adolescents to offer significant health benefits and mitigate health risks. Physical fitness and sports practice seem to improve oxidative stress (OS) status during childhood. However, to our knowledge, there are no data regarding the influence of objectively-measured physical activity (PA) and sedentarism on OS status in children and adolescents. The present study aimed to evaluate the influence of moderate and vigorous PA and sedentarism on OS and plasma total antioxidant capacity (TAC) in a selected Spanish population of 216 children and adolescents from the GENOBOX study. PA (light, moderate, and vigorous) and sedentarism (i.e., sedentary time (ST)) were measured by accelerometry. A Physical Activity-Sedentarism Score (PASS) was developed integrating moderate and vigorous PA and ST levels. Urinary 8-hydroxy-2′-deoxyguanosine (8-OHdG) and isoprostane F2α (F_2_-IsoPs), as markers of OS, were determined by ELISA; and TAC was estimated by colorimetry using an antioxidant kit. A higher PASS was associated with lower plasma TAC and urinary 8-OHdG and F_2_-IsoPs, showing a better redox profile. Reduced OS markers (8-OHdG and F_2_-IsoPs) in children with higher PASS may diminish the need of maintaining high concentrations of antioxidants in plasma during rest to achieve redox homeostasis.

## 1. Introduction

Physical activity improves cardiorespiratory fitness and strengthens the musculoskeletal system, helping to maintain proper body composition, both in children and adolescents [1,2]. Physical activity practice also seems to reduce lipid peroxidation and improve the antioxidant defense system, resulting in the maintenance of redox homeostasis [3].

The concept of “oxidative stress” has been defined as “an imbalance between oxidants and antioxidants in favor of the oxidants, leading to a disruption of redox signaling and molecular damage” [4]. Urinary isoprostanes and 8-hydroxy-2′-deoxyguanosine (8-OHdG), among other body-oxidized compounds, are well-recognized biomarkers of oxidative stress, which have been found to be increased in several situations, including obesity, type 2 diabetes, and cardiovascular diseases [5]. F2-8-iso-prostaglandin F2α, also known as isoprostane F_2_α (F_2_-IsoPs), is generated by free radical-induced peroxidation of arachidonic acid and is currently regarded as one of the most reliable biomarkers of in vivo oxidative stress [6]. In addition, 8-OHdG is considered to be a biomarker of generalized cellular oxidative stress [7]; it can be easily determined in urine with good reproducibility and recovery of untimed samples [8].

Two subsystems integrate the antioxidant defense system: the first is related to the activity of several enzymes such as glutathione peroxidases, glutathione reductase, glutathione S-transferase, superoxide dismutases, and catalase; the second is formed by non-enzymatic antioxidants such as uric acid, tocopherols, ascorbate, glutathione and ubiquinone [9]. The non-enzymatic component can be chemically measured in blood plasma by facing it to several oxidizing substances, obtaining the non-enzymatic antioxidant capacity (NEAC), total antioxidant status, or, henceforth, total antioxidant capacity (TAC). The use of indices of global redox status, such as plasma TAC, may be more appropriate than the comparison of single biomarkers to evaluate oxidative stress. In this context, TAC seems a useful parameter for assessing the global redox status in children and adolescents [10]. 

Regardless of dietary modifications, physical activity interventions seem to improve body mass index in children and adolescents [11,12]. Similarly, the practice of physical activity, both in adults and children, has been associated with an increase in antioxidants and a reduction of pro-oxidants [13]. Additionally, obesity has been related to an imbalanced redox status [14]. However, it is unclear whether the effect of exercise on redox status is mediated or not by changes in body weight status.

The effect of acute and chronic physical activity practice on oxidative stress responses in children and adolescents has been recently reviewed [15]. Acute exercise seems to induce a relevant, but transient, increase in markers of oxidative stress. In contrast, regular exercise appears to be associated with increased antioxidants and reduced systemic oxidative biomarkers, even independently of body weight status. However, the studies included in that review are heterogeneous in terms of the type of exercise, intensity, and time of physical activity practiced [15].

The World Health Organization (WHO) and other international institutions have recommended that children and adolescents should perform at least an average of 60 min per day of moderate to vigorous-intensity, mostly aerobic activities across the week, in order to achieve several positive outcomes regarding cardiovascular, metabolic, and musculoskeletal health [16]. However, the impact of physical activity, especially in terms of time and intensity, on redox status, has not been accurately described for children and adolescents. In fact, to our knowledge, evaluation of the redox status of children according to objectively-measured physical activity by accelerometry has not been investigated. 

We hypothesized that physical activity practice, especially moderate-to-vigorous physical activity (MVPA), and sedentary time could be related to higher plasma levels of TAC in children and adolescents, showing a better global redox status. For this purpose, we aimed to compare plasma levels of TAC and biomarkers of oxidative stress (urinary F_2_-IsoPs and 8-OHdG) according to a Physical Activity-Sedentarism Score (PASS), characterized by moderate and vigorous physical activity and sedentary time measured by accelerometry, in a cross-sectional sample of Spanish children from the GENOBOX study.

## 2. Materials and Methods

### 2.1. Population

The present work was part of the GENOBOX study. GENOBOX is a case-control, multicenter study carried out in a total of 1444 children (706 males and 738 females), aged 3 to 17 years Spanish children during 2012–2015. Detailed inclusion and exclusion criteria as well as informed consent and approval by the local Ethics Committees of the three Spanish Hospitals (Hospital Universitario Reina Sofía, Córdoba; Hospital Clínico Universitario, Santiago de Compostela; and Hospital Clínico Universitario Lozano Blesa, Zaragoza, Spain; Code IDs: Córdoba 01/2017, Santiago 2011/198, Zaragoza 12/2010) where children were recruited have been reported elsewhere [14]. A subsample of 216 children (111 boys) was selected based on the following inclusion criteria for the present study: Caucasian children and adolescents aged 6 to 14 with body composition measured by bioelectrical impedance analysis, valid blood measurements including sex hormones (follicle-stimulating hormone, luteinizing hormone, testosterone in boys, and estradiol in girls), and accurate data from an accelerometry standardized protocol, as well as measured plasma levels of TAC and urinary F_2_-IsoPs and 8-OHdG. Within the subsample of 216 subjects, a subgroup of 74 children (33 boys) also had measures of plasma carotenes, retinol, and tocopherols. 

### 2.2. Clinical and Anthropometric Examination

Medical history and a physical exam including the evaluation of sexual maturity according to Tanner’s five-stages were assessed and confirmed with sexual hormone measurements. Anthropometric measurements were taken by a single examiner, and details have been previously reported [17]. 

### 2.3. Blood Sampling

Blood samples were drawn from the antecubital vein between 08:00 and 09:30 h after an overnight fast. Routine blood tests (Glucose (CV = 3.0%), plasma insulin (CV = 2.6%), follicle-stimulant hormone (CV = 3.6%); luteinizing hormone (CV = 3.1%), testosterone (CV = 2%), and estradiol (CV = 1.8%) were measured as previously reported using automated analyzers [17] The homeostasis model assessment of insulin resistance (HOMA-IR) was calculated based on the published equation: HOMA-IR = fasting glucose (mmol) × fasting insulin (mU/mL)/22.5 [18]. 

Plasma TAC was determined by colorimetry using a commercial antioxidant assay kit (Cat no. 709001, Cayman Chemical, Ann Arbor, MI, USA). Urinary F_2_-IsoPs was determined by using a commercial competitive ELISA kit (EA85 Oxford Biomedical Research, Oxford, MI, USA) (CV): 14.13%). Urinary 8-OHdG was also determined by using a commercial competitive ELISA kit (KOG-200S/E JaICA, Fukuroi, Japan) (CV: 5.73%). The concentrations were normalized by urinary creatinine and expressed in ng/mg of creatinine. Creatinine concentration in urine samples was determined with a colorimetric kit (Ref. 1001115, Spinreact, Barcelona, Spain) (CV: 2.89%). Measurement of plasma concentrations of retinol, carotenes, and tocopherols were determined after extraction with 1-propanol by ultra-high-pressure liquid chromatography coupled to mass spectrometry UHPLC-MS as reported elsewhere [14].

### 2.4. Accelerometry

ActiGraph GT3X and GT3X+ accelerometers (ActiGraph; Pensacola, FL, USA) were used to assess physical activity levels in this study. Accelerometers were placed over the right iliac crest and held in place using an adjustable elastic belt for 24 h a day with a minimum of 8 h of monitoring per day for at least 3 days (at least one weekend day). It was programmed for 15 epochs (period of 15 s), as previously recommended [19]. Accelerometry data were processed using the Actilife v6.13.3 program (ActiGraph; Pensacola, FL, USA) replacing, as a missing data code before further analysis, all negative counts and periods of 20 min or more of consecutive zero counts were replaced [20]. The output generated by the ActiGraph GT3X+ included the total volume of physical activity and each physical activity intensity as defined by the cut-points of counts per minute (CPMs) based in the classification by Evenson et al. [21]. 

### 2.5. Statistical Analysis

All continuous variables were tested for normality using the Shapiro–Wilk and Kolmogorov tests; the variables following a non-normal distribution were square-root (TAC, 8-OHdG, F_2_-IsoPs, fat body mass (FM), moderate and vigorous physical activity) or logarithm transformed (sedentary time). The homogeneity of variances was estimated using Levene’s test. Differences between pre-pubertal and pubertal children were analyzed by two-independent-sample t-tests or Mann–Whitney U tests. χ^2^ tests were applied to categorical variables expressed in percentage.

Principal component analysis (PCA) was performed to investigate the relationships among body mass index, body composition, peripheral tissue insulin resistance—as a risk feature of metabolic syndrome—physical activity levels, and oxidative stress and TAC in the 210 children. Extraction of the initial set of uncorrelated components was accomplished with the principal factor method, and then Varimax orthogonal rotation of components was used to facilitate interpretation. High loading values indicate a stronger relationship between a factor and an observed variable. Factor loadings lower than 0.359 (critical factor, *p* < 0.001) revealed marginal correlations.

To estimate the overall physical activity and sedentarism levels, a composite activity score of sedentary time, moderate and vigorous intensities was calculated (i.e., PASS). For this purpose, quartiles of each variable were designed, being the minutes of moderate and vigorous physical activity higher for the fourth quartile compared to the first one, and the opposite for sedentary time. Each subject obtained 1 to 4 points according to the quartile of each variable (e.g., 1 point for being in Q1 for moderate physical activity or 4 points for being in Q4 for sedentary time). By summing the points of the three variables, the PASS was obtained for each subject. The PASS ranged from 3 (all variables in the first quartile) to 12 (all variables in the fourth quartile); a higher score indicated a higher active habit. Based on this score, four groups of PASS were established: very low active (VLA; a score of 3), low active (LA; score from 4 to 6), moderately active (MA; score from 7 to 9), and high active (HA; score from 10–12). MANOVA was used to test the difference between the four groups across several outcome variables/outcomes simultaneously and Box’s test looks at the assumption of equal covariance matrices. Pillai’s trace value lower than 0.05 was considered statistically significant for the MANOVA test. Differences between the four groups based on the PASS were later analyzed using univariant ANCOVA (UNIANCOVA), adjusting for age and/or body mass index (BMI) z-score; pairwise differences were assessed by post hoc analyses to determine differences between experimental groups. For those variables not following normality, a Kruskal–Wallis test was used to evaluate differences between groups. Values in the descriptive tables and results are expressed as means and standard deviations. Differences were considered significant when *p* < 0.05. All statistical procedures were conducted using SPSS (IBM SPSS Statistics, Version 25.0. Armonk, NY, USA).

## 3. Results

General demographic, anthropometric, physical activity, and peripheral insulin resistance variables in prepubertal and pubertal selected children within the GENOBOX study are shown in Table 1. Within the 216 subjects, 105 were at prepubertal and 111 at the pubertal stage. There were no differences between prepubertal and pubertal groups regarding BMI z-score and percentages of FM and fat free mass (FFM). No differences were also found for moderate physical activity, but it was closed to statistical significance. At the same time, HOMA-IR and sedentary time were significantly higher for those at pubertal status.

From the eight items included in the PCA (physical activity, body composition, insulin resistance, oxidative stress, and total plasma antioxidant capacity), three principal components were extracted (Table 2), which explained 64.4% of the total variance (30% of the variance was explained by the first factor, an additional 21% by the second factor, and another 13% by the third factor) (Table 3). The first principal component, termed “metabolic risk” showed a positive correlation between HOMA-IR, FM, and FFM, and humble correlations for sedentary time (negative) and MVPA (positive). The second component, termed “oxidative stress”, included correlations among urinary F_2_-IsoPs, 8-OHdG, and sedentary time. The third component named “physical activity” included a positive correlation between FFM and MVPA, and negative with sedentary time. TAC did not reach the critical value to be included in any of the components.

**Figure 1:**
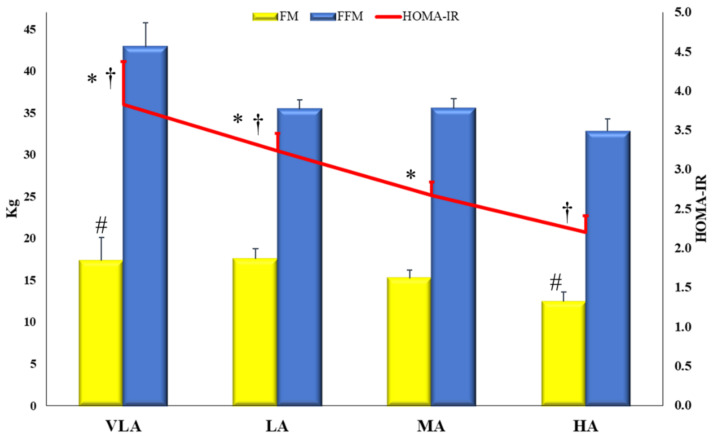
Fat mass, fat-free mass and HOMA-IR according to Physical Activity-Sedentarism Score (PASS) groups in Spanish children from the GENOBOX study. FM: fat mass; FFM: fat-free mass; HOMA-IR: homeostasis model assessment-insulin resistance; VLA: very low active; LA: low active; MA: moderately active; HA: high active. Differences between PASS levels were analyzed using one-way ANCOVA, adjusting for age and/or BMI z-score; pairwise differences were assessed by post hoc analyses to determine differences between experimental groups. For those variables not following normality, a Kruskal–Wallis test was used to evaluate differences between groups. FM: # Differences between the HA and VLA of (*p* = 0.003). HOMA-IR (UNIANCOVA, *p*=0.005): † Differences between HA vs. LA and VLA (*p* = 0.018 and *p* = 0.010, respectively); * Differences between A vs. LA and VLA (*p* = 0.014 and *p* = 0.010, respectively). *p* for trend for HOMA-IR (*p* = 0.001) and FM (*p* = 0.044).

**Figure 2:**
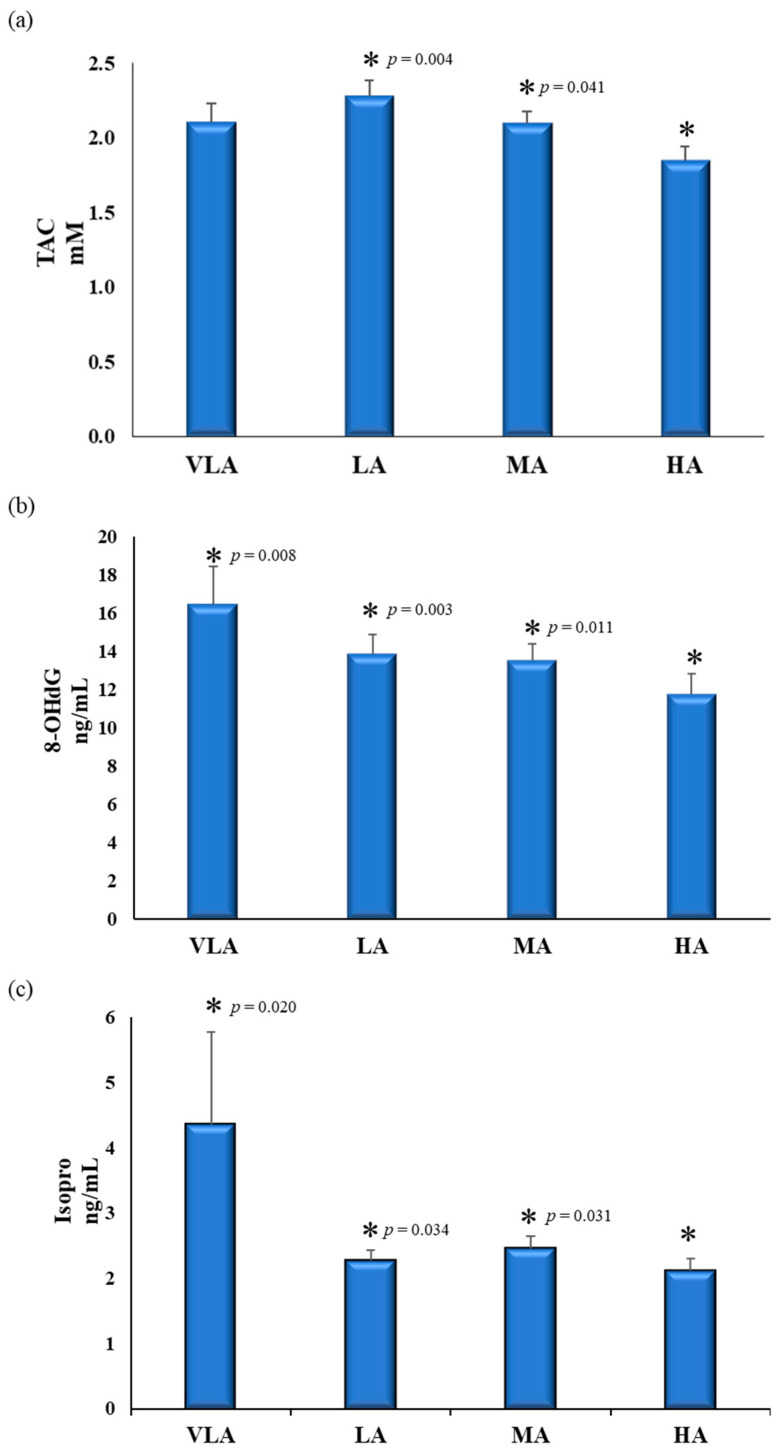
(**a**)Total plasma antioxidant capacity, (**b**) urinary 8-hydroxy-2’-deoxyguanosine and (**c**) F_2α_-Isoprostanes by Physical Activity-Sedentarism Score (PASS) groups in Spanish children from the GENOBOX study. TAC: total plasma antioxidant capacity; 8-OHdG: 8-hydroxy-2’-deoxyguanosine; F_2_-IsoPs: F_2α_-isoprostanes; PA: physical activity; VLA: very low active; LA: low active; MA: moderately active; HA: high active. Differences between PASS levels were analyzed using one-way ANCOVA, adjusting for age and/or BMI z-score; pairwise differences were assessed by post hoc analyses to determine differences between experimental groups. For those variables not following normality, a Kruskal–Wallis test was used to evaluate differences between groups. * Differences with HA score. *p* for trend for TAC (*p* = 0.020).

**Figure 3:**
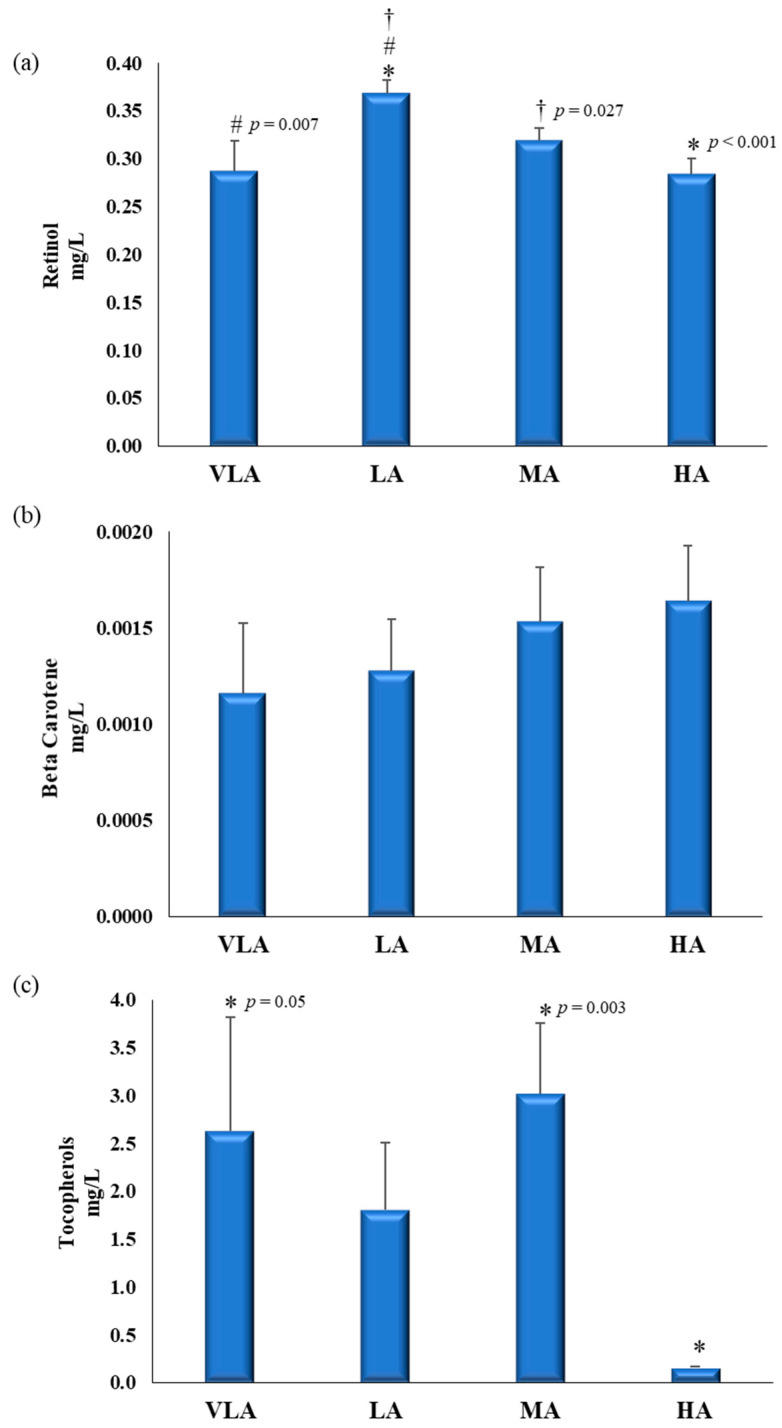
(**a**) Plasma retinol, (**b**) beta-carotene, and (**c**) tocopherols levels according to the Physical-Activity-Sedentarism Score (PASS) levels in Spanish children from the GENOBOX study. PA: physical activity; VLA: very low active; LA: low active; MA: moderately active; HA: high active. Differences between PASS levels were analyzed using one-way ANCOVA, adjusting for age and/or BMI z-score; pairwise differences were assessed by post hoc analyses to determine differences between experimental groups. For those variables not following normality, a Kruskal–Wallis test was used to evaluate differences between groups. * Differences with HA; # Differences with VLA; † Differences MA. *p* for trend for retinol (*p* = 0.043) and tocopherols (*p* = 0.040).

The differences between PASS levels for the variables included in the PCA are presented in Table 4 and Figure 1, Figure 2 and Figure 3. Children with a higher PASS allocated in the high active group had a lower BMI z-score than those in the moderately active and low active groups. 

Figure 1 depicts the differences between the PASS levels for the variables with a higher factor loading of the “metabolic risk” component (FM, FFM, and HOMA-IR). Children in the high active group showed the lowest levels of FM and HOMA-IR. No differences were found between groups for FFM. 

The influence of PASS on redox status, assessed by plasma TAC, and urinary F_2_-IsoPs and 8-OHdG, is represented in Figure 2. TAC’s plasma level was lower for those children in the high active group compared to those in the moderately active and low active groups (UNIANCOVA *p* = 0.035, Figure 2a). Regarding the evaluation of the oxidative stress status, children in the high active group showed lower urine levels of 8-OHdG (Figure 2b) and F_2_-IsoPs (Figure 2c) than the rest of the groups (UNIANCOVA *p* = 0.005 and *p* = 0.036, respectively).

Finally, the plasma concentration of some components of the non-enzymatic antioxidant defense system, retinol, beta-carotene, and tocopherols were measured in a subgroup of our study. Differences in the plasma levels of these biomarkers, according to the PASS groups, are presented in Figure 3. Children in the low active group showed the highest values of retinol (UNIANCOVA, *p* = 0.002) compared to those in other groups. Regarding tocopherols (UNIANCOVA, *p* = 0.022), children in the high active group showed lower levels compared with children with very low active and moderately active groups.

## 4. Discussion

The influence of objectively measured physical activity and sedentary time on redox status of children and adolescents has been scarcely described so far. In the present analysis, a high PASS, characterized by a high time spent on moderate and vigorous physical activity, along with a low sedentary time, was associated with lower levels of plasma TAC, and urinary F_2_-IsoPs and 8-OHdG and, therefore, to better redox status. 

Differences in the redox profile (oxidants and antioxidants) of children and adolescents according to their physical fitness, sports practice, and physical activity collected by questionnaires have been previously reported [15,22,23]. However, since WHO has recommended to “practice at least an average of 60 min per day of moderate- to vigorous-intensity physical activity across the week” and to “limit the amount of sedentary time, particularly the amount of recreational screen time”, we noticed that there are no data about the influence of physical activity, in terms of duration and intensity, and sedentarism on redox status [16]. In this context, our study showed that high active children, who performed 76 min of MVPA on average and spent significantly less time being sedentary, presented lower levels of plasma TAC, urinary F_2_-IsoPs and 8-OHdG than moderately active children who performed a mean of MVPA of 55 min/day, close to the international recommendation. These results agree with the statements of the WHO about physical activity practice and sedentary time limitation in children and adolescents, but also suggest the possible benefit on redox status of increased MVPA practice. 

In our study, contrary to what we expected, children with a higher PASS, especially high active children, showed the lowest plasma TAC concentrations. There are several possible factors that could explain this finding. On one hand, the effect of physical activity practice on redox status is different for acute or chronic exercise. Globally, acute exercise produces a transient increase in both pro-oxidant and antioxidant biomarkers, while a regular physical activity practice improves the antioxidant defense system and reduces the systemic levels of oxidative stress markers [15]. Focusing on TAC plasma levels, they seem to increase in response to acute exercise, while the effect of chronic exercise on these concentrations remains controversial [15,24,25]. Regarding the oxidative stress biomarkers measured in our study, urinary 8 F_2_-IsoPs and 8-OHdG, the available evidence of the long-term effect of regular physical activity on these parameters is scarce [26,27]. Nasca et al. reported an increase in urinary F_2_-IsoPs concentrations after a 5-week exercise program in scholars [26]. In contrast, no significant changes in urinary F_2_-IsoPs concentrations were found after a 3-month exercise program in adolescent girls [27]. These results are controversial and none of these studies measured biomarkers of the antioxidant defense system to evaluate de redox status. However, in our study, we also found that those children with a high PASS and low TAC plasma concentration also showed the lowest urine concentrations of 8-OHdG and F_2_-IsoPs. In this context, we hypothesize that concomitantly reduced oxidative stress markers in children with a high PASS may explain a diminished need of maintaining high concentrations of antioxidants in plasma during rest to achieve redox homeostasis. Regular practice of physical activity may reduce basal levels of oxidative stress biomarkers, improve the response of the antioxidant defense system to a stress situation, or even both. In this way, it would be interesting to evaluate the differences in the plasma levels of TAC in response to an acute planned exercise between groups with different PASS. 

On the other hand, body composition (FM and FFM) is also related to differences in the redox status of children. Obesity and its associated comorbidities have been linked to imbalanced redox homeostasis both in adults and children [14,28]. In fact, plasma concentrations of TAC seem to be lower in children with obesity when compared to normal-weight peers [24,29,30]. However, Ruperez et al. [14] recently described different plasma TAC concentrations according to pubertal status. Thus, obese children at the prepubertal stage had lower TAC and pubertal children higher TAC than controls [14]. In this context, available evidence suggests that obesity may negatively affect the exercise-related antioxidant responses to acute exercise [15,24]. While the regular practice of physical activity seems to improve both, body composition [11,12] and oxidative stress status [13,15], as much in children as in adults. In order to shed light on the relationships between these variables, the PCA performed in the present study showed that body composition was associated with MVPA and sedentary time (first component: metabolic risk). In contrast, oxidative stress biomarkers were associated with sedentary time (second component: oxidative stress) (Table 3). In addition, a higher score of PASS was associated with lower FM and HOMA-IR, but also with lower levels of TAC, urinary 8-OHdG and F_2_-IsoPs, even after age- and BMI z-score-adjustments. These data suggest that regular practice of physical activity can influence oxidative stress improvement in both ways, indirectly (through changes in body composition) and directly. 

Lastly, pubertal status has been related to differences in the redox response to exercise. Available evidence suggests that the transition from childhood to adolescence may promote a maturation of pro-oxidant and anti-oxidant mechanisms associated with the activation of somatotropic and gonadal axes [15]. Recently, Chaki et al. [31] compared the redox status, from baseline and after a high-intensity exercise, between sedentary pre- and post-pubertal boys. They found in both baseline and post-exercise that pro-oxidant and anti-oxidant biomarkers were higher for those with pubertal maturation. They suggested post-pubertal boys may face to oxidative stress more efficiently than their prepubertal peers [31]. Following the same trend, pubertal children of our sample exhibited higher levels of TAC than those who were prepubertal and the relationship between TAC and PASS levels remained negative for both groups but attenuated for pubertal children (data not shown). 

Regarding sedentary time, there are no data about its effect on oxidative stress status in children and adolescents. Previous reports evaluating the influence of sedentarism are only focused in sports practitioners. In these studies, higher plasma TAC levels have been reported in professional sports practitioners (basketball players, judokas, kayakers, and canoeists) than in sedentary controls, both at rest and after exercise [32,33,34]. However, these professional sports practitioners also showed higher levels of OS biomarkers than sedentary controls, both at rest and after exercise.

TAC levels were lower in high active group compared with low active group. However, the concentrations of retinol, beta-carotene, and tocopherols, which contribute to determining TAC, had a different trend. The reason for these apparently contradictory results may be because plasma TAC is influenced not only by liposoluble antioxidants, namely α-tocopherol, β-carotene, which are mainly located in lipoproteins, and retinol, which is bounded to retinol-binding protein but for plasma proteins, notably albumin and other thiols rich proteins, urate and ascorbate. The contribution to TAC from urate has been reported to be (35–65%) and plasma proteins (10–50%), while ascorbate (0–24%) and tocopherol (5–10%) [35]. 

## 5. Strengths and Limitations

To our knowledge, this is the first study evaluating the influence of objectively measured physical activity and low active by accelerometry on oxidative stress status in children and adolescents. In addition, most previous studies analyzing redox status of children were focused on obesity and its comorbidities, while the role of physical activity in this relationship had not been adequately investigated. By last, the PASS, which integrates the main components of the WHO recommendations for physical activity and sedentary behavior, showed as a useful parameter to evaluate the health benefits of increased physical activity and reduced sedentary time. 

In contrast, our study presents several limitations. First, it is not a longitudinal or intervention study, so we can only describe results and generate hypothesis of the relationship between physical activity and oxidative stress status. Second, among the multiple oxidative stress and antioxidant defense system biomarkers, the most appropriate parameters to evaluate redox status in children and adolescents have not been well established yet. The measurement of TAC is useful for evaluating the redox profile, but it is only a part of the antioxidant defense system. In the same way, F_2_-IsoPs and 8-OHdG are two of the most common biomarkers of oxidative stress. In this context, we measured plasma TAC and urinary F_2_-IsoPs and 8-OHdG in an acceptable sample of children and adolescents, while the sample size was substantially reduced when evaluating non-enzymatic biomarkers of oxidative stress (retinol, beta-carotene, and tocopherols) due to the limited availability of blood samples. In this context, due to the variance of plasma TAC and oxidative stress biomarkers, it would be necessary a higher sample size to validate the results of the present work. Furthermore, some other factors not taken into account, such as nutrition or physical fitness, could also mediate the relationship between PA and oxidative stress status. 

## 6. Conclusions

In conclusion, a high physical-activity-sedentarism score characterized by a high time devoted to moderate and vigorous physical activity, along with a low sedentary time, was associated with lower plasma TAC levels and reduced urinary F_2_-IsoPs and 8-OHdG concentrations, contributing to a better redox profile. Future research should evaluate if these active children perform a better redox response to exercise than those who are less active.

## Figures and Tables

**Table 1:** General demographic, anthropometric, physical activity, and peripheral insulin resistance variables in prepubertal and pubertal Spanish children from the GENOBOX study.

Variables	All Participants (216)	Prepubertal (105)	Pubertal (111)	*p*-Value
Age (years)	10.8 ± 2.2	9.4 ± 1.7	12.1 ± 1.7	<0.001
Weight (kg)	50.3 ± 17.3	41.8 ± 12	58.2 ± 17.9	<0.001
Height (m)	1.47 ± 0.13	1.38 ± 0.10	1.55 ± 0.10	<0.001
BMI (kg/m^2^)	22.9 ± 5.2	21.7 ± 4.5	23.9 ± 5.6	0.003
BMI z-score	1.15 ± 2.14	1.11 ± 2.38	1.15 ± 2.18	0.886
FM (kg)	15.2 ± 9.3	12.8 ± 7	17.3 ± 10.6	0.001
FM (%)	27.8 ± 9.9	27.9 ± 9.3	27.6 ± 10.4	0.836
FFM (kg)	35.1 ± 10.2	29.4 ± 6.4	40.4 ± 10.3	<0.001
FFM (%)	71.6 ± 11.1	71.5 ± 10.4	71.7 ± 11.8	0.887
Normal weight (%)	34.9	14.9	20.2	0.342 *
Overweight (%)	23	11.5	11.5	0.342 *
Obesity (%)	42.1	22.6	19.2	0.342 *
HOMA-IR	2.91 ± 1.81	2.32 ± 1.54	3.40 ± 1.83	<0.001
ST (min/d)	482 ± 97	467 ± 103	495 ± 89	0.018
PA Moderate (min/d)	38 ± 14	40 ± 13	36 ± 15	0.050
PA Vigorous (min/d)	15 ± 10	14 ± 8	16 ± 11	0.251
MVPA (min/d)	53 ± 21	54 ± 20	52 ± 23	0.494

BMI: body mass index; FM: fat mass; FFM: fat-free mass; HOMA-IR: homeostasis model assessment-insulin resistance; ST: sedentary time; PA: physical activity; MVPA: mean moderate-vigorous physical activity. Data are shown as mean ± SD. Student’s *t*-test for parametric analysis and U de Mann–Whitney for non-parametric analysis was used to compare variables between prepubertal and pubertal stages. * represents *p* value for Chi-square test.

**Table 2:** Principal component analysis for the GENOBOX study extracted from physical activity, body composition, peripheral tissue insulin resistance, oxidative stress, and total plasma antioxidant capacity variables.

Variables	Component matrix ^a^		
	Factor *		
	Metabolic Risk	Oxidative Stress	Physical Activity
HOMA-IR	0.831		
FM (kg)	0.804		
FFM (kg)	0.798		0.388
TAC (mM)	0.356		
8-OHdG (ng/mL)		0.859	
F_2_-IsoPs (ng/mL)		0.831	
MVPA (min/d)	−0.377		0.771
ST (min/d)	0.366	0.387	−0.422

HOMA-IR: homeostasis model assessment-insulin resistance; FM: fat mass; FFM: fat-free mass; TAC: plasma total antioxidant capacity; 8-OHdG: urinary 8-hydroxy-2′-deoxyguanosine; F_2_-IsoPs: urinary F2α-isoprostanes; MVPA: mean moderate-vigorous physical activity; ST: sedentary time. **^a^** Extraction of the initial set of uncorrelated components was accomplished with the principal factor method, and then the Varimax orthogonal rotation of components was used to facilitate interpretation. The number of components retained was based on Scree plot analysis and eigenvalues greater than 1 (with the components accounting for more of the total variance than any single variable). * Factor loading is the product-moment correlation (a measure of linear association) between an observed variable and an underlying factor. A significant loading factor was defined as a value greater than 0.359 (*p* < 0.01).

**Table 3:** Eigen values and percentages of variance associated with each linear component (factor) before extraction, after extraction, and after rotation, in the principal component analysis for the GENOBOX study of children relating physical activity, body composition, and peripheral tissue insulin resistance to risk factors for oxidative stress and total plasma antioxidant capacity.

Component	Total Variance Explained								
	Initial Eigenvalues			Sums of Loads Squared from Extraction			Sums of Loads Squared of Rotation		
	Total	% of variance	% Accumulated	Total	% of Variance	% Accumulated	Total	% of Variance	% Accumulated
1	2.402	30.029	30.029	2.402	30.029	30.029	2.232	27.901	27.901
2	1.694	21.175	51.204	1.694	21.175	51.204	1.634	20.424	48.325
3	1.063	13.293	64.496	1.063	13.293	64.496	1.294	16.171	64.496

The first value in the row gives the proportion of variance (the degree of spread in the data set) explained by body composition and tissue peripheral resistance; the second value, the proportion explained by oxidative stress; and the third value, the proportion explained by the level of MVPA and sedentarism.

**Table 4:** Differences in body mass index (BMI) z-score, moderate-vigorous physical activity and sedentary time between groups of Physical Activity-Sedentarism Score (PASS) of the Spanish children from the GENOBOX study.

Variables	Physical Activity-Sedentarism Score Levels				*p*-Value	*p*-Value for Trend
	Very Low Active (12)	Low Active (65)	Moderate Active (82)	High Active (57)		
BMI z-score	1.25 ± 1.4 ^a,b^	1.57 ± 2.1 ^a,b^	1.41 ± 1.9 ^a,b^	0.51 ± 2.9 ^a,c^	0.046	0.029
PASS	3 ^a^	5.27 ± 0.78 ^b^	8 ± 0.84 ^c^	10.88 ± 0.81 ^d^	<0.001	<0.001
MVPA (min/d)	22 ± 5 ^a^	34 ± 11 ^b^	55 ± 12 ^c^	76 ± 17 ^d^	<0.001	<0.001
ST (min/d)	601 ± 70 ^a^	517 ± 92 ^b^	471 ± 95 ^c^	397 ± 54 ^d^	<0.001	<0.001

BMI: body mass index; PASS: Physical Activity-Sedentarism Score; PA: physical activity; MVPA: moderate-vigorous physical activity; ST: Sedentary time. Data are shown as mean ± SD. One-way ANOVA test was used to compare variables among the different PASS levels. No matching superscript letters (a, b, c, d) indicate significant differences (*p* < 0.05) by pairwise post hoc test adjusted for age and/or BMI z-score to determine which experimental groups differed from each other.

## Data Availability

The data presented in this study are available on request from the corresponding author. The data are not publicly available due to privacy restrictions.
